# Advances in Peripheral Nerve Block Techniques and Clinical Strategies for Their Implementation Following Total Knee Arthroplasty: A Narrative Review

**DOI:** 10.3390/jcm15051957

**Published:** 2026-03-04

**Authors:** Vendhan Ramanujam, Justin Bessette, Jasper Yeh, Yash Shah, Bijan Moazezi, Mark C. Kendall

**Affiliations:** 1 Department of Anesthesiology, Warren Alpert Medical School of Brown University, Providence, RI 02903, USA; vendhan_ramanujam@brown.edu (V.R.); yshah@brownhealth.org (Y.S.);; 2 The Warren Alpert Medical School of Brown University, Providence, RI 02903, USA

**Keywords:** total knee arthroplasty, peripheral regional anesthesia, multimodal analgesia, postoperative outcomes, motor-sparing regional anesthesia

## Abstract

Total knee arthroplasty (TKA) is one of the most performed surgical procedures in the United States and is often associated with moderate to severe postoperative pain. Multimodal postoperative analgesia following TKA is essential for optimizing postoperative recovery and enabling early postoperative mobilization. Regional anesthesia using ultrasound-guided peripheral nerve blocks plays an important part in perioperative pain management by targeting the femoral, obturator, and sciatic nerves of the knee joint. A variety of peripheral nerve block techniques have been described, which can be classified as either motor-blocking or motor-sparing techniques. Traditional motor-blocking regional anesthesia techniques, such as femoral and sciatic nerve blocks, provide excellent analgesia but can result in significant quadriceps weakness that delays ambulation after TKA. Motor-sparing regional anesthesia techniques, including the adductor canal block, iPACK block, and genicular nerve block, are becoming more widely used in enhanced postoperative recovery protocols for outpatient and short-stay inpatient TKAs. The peripheral nerve block technique can be selected according to the type of surgical procedure, the planned length of stay, rehabilitation goals, and patient comorbidities. Multiple peripheral nerve blocks provide better analgesia than single-injection blocks, and continuous catheter techniques are used for prolonging analgesia in select patients. An individualized multimodal regional anesthesia approach should be utilized to maximize analgesia after TKA to optimize postoperative outcomes. We present a narrative review of peripheral nerve block techniques and strategies for their use following inpatient or outpatient TKA.

## 1. Introduction

Total knee arthroplasty (TKA) is among the most performed surgical procedures in the United States, with more than 700,000 operations performed annually [1]. As a major joint surgery, TKA is associated with considerable postoperative pain, with more than half of patients experiencing moderate to severe postoperative pain [2]. Therefore, multimodal analgesia, in which various approaches are utilized to target multiple pain pathways, plays a key role in effectively managing pain in these patients to facilitate their smooth recovery. Among the different approaches available, regional anesthesia using peripheral nerve blocks is a key strategy for both intraoperative and postoperative pain control. Local anesthetics administered near the nerve interrupt the transmission of pain signals from the knee to the brain, thereby providing effective pain relief. Ultrasound-guided (USG) nerve block techniques have become the modern standard and are commonly used due to their advantages, such as greater precision, improved safety, and higher success rates. The innervation of the knee joint originates from the lumbar and sacral plexus through the femoral, obturator, and sciatic nerves [3]. These nerves branch into 10 nerve branches, which further divide into at least 17 smaller branches that ultimately supply the knee joint capsule [4]. Multiple USG nerve block techniques targeting either sensory nerves alone or both sensory and motor blockade have emerged over the years, with the latter often resulting in some degree of weakness that can impact the speed of recovery and rehabilitation following knee replacement surgery [5]. In this review, we discuss the various ultrasound-guided peripheral nerve blocks currently used for pain management in the context of TKA. We also explore potential nerve block combinations tailored to individualized postoperative care.

## 2. Overview

This narrative review provides an overview of peripheral nerve blocks and periarticular analgesia for total knee arthroplasty, with a focus on analgesic efficacy, motor-sparing effects, and their role within multimodal postoperative pain management. Both traditional motor-blocking techniques and newer motor-sparing approaches, including adductor canal, iPACK, and genicular nerve blocks, are discussed in relation to early mobilization and functional recovery. A comprehensive, non-systematic search was conducted to provide an overview of current best practices. No formal risk of bias assessment or meta-analysis was performed. Studies were included based on their relevance to clinical decision-making and pain management strategies.

## 3. Peripheral Nerve Block Techniques

### 3.1. Lumbar Plexus Block

The lumbar plexus block (LPB) was originally described by Winnie et al. in 1973 as a “three-in-one” technique targeting the femoral, lateral femoral cutaneous, and obturator nerves [6]. The lumbar plexus, formed by contributions from L1–L4 nerve roots, gives rise to six peripheral nerves, three of which innervate regions superior to the knee and are not directly relevant for knee surgery. The femoral nerve, originating from the posterior divisions of L2–L4, is the largest branch and contributes to the patellar plexus through its intermediate branch. The lateral femoral cutaneous nerve arises from the posterior division of L2–L3 and provides purely sensory innervation to the knee through the distal division of its anterior branch, which also contributes to the patellar plexus. The final nerve—the obturator nerve—originates from the anterior divisions of L2–L4, providing motor and sensory innervation to the anterior thigh and contributing articular branches to the posterior aspect of the knee [7].

**Figure 1:**
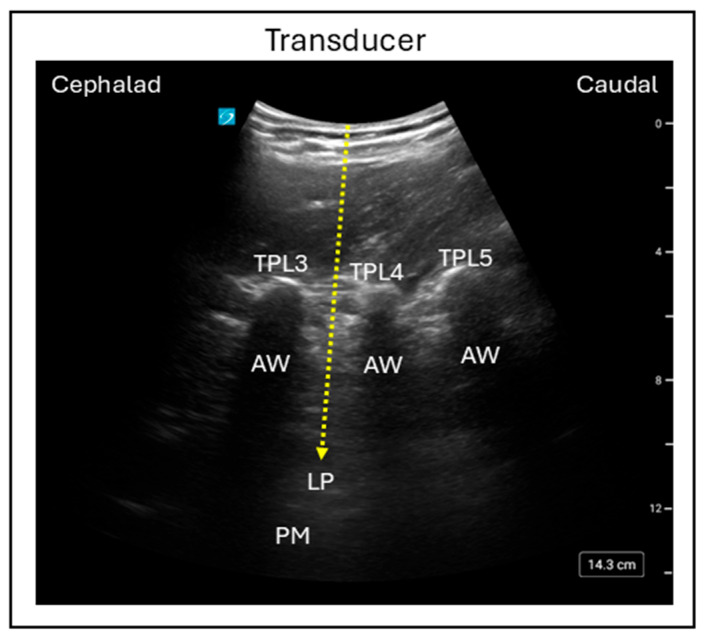
Ultrasound image demonstrating the in-plane trident lumbar plexus block technique using the posterior approach. Dashed line = Needle trajectory; TPL3 = Transverse process lumbar 3; TPL4 = Transverse process lumbar 4; TPL5 = Transverse process lumbar 5; AW = Acoustic window; LP = Lumbar plexus; PM = Psoas muscle.

As the lumbar plexus lies in a deep anatomical plane, ultrasound guidance has become the preferred technique for performing lumbar plexus block due to its capacity to improve visualization, accuracy, and patient safety. The lumber plexus block has been described using both anterior and posterior approaches; however, the anterior approach frequently provides incomplete coverage of the obturator and lateral femoral cutaneous nerves. In contrast, the posterior approach reliably blocks all three target nerves, making it the preferred technique in clinical practice [8]. Among the posterior techniques, the lumbar ultrasound trident technique and the Shamrock technique are most commonly used. In the trident technique, the patient sits upright while a low-frequency curvilinear transducer is positioned a few centimeters lateral to the midline in a sagittal orientation, allowing for visualization of the lumbar paravertebral region. The lumbar transverse processes appear as hyperechoic reflections with acoustic windows distal to them and, when the transducer is positioned over L2–L4, a series of acoustic shadows resembling a “trident” sign can be observed. Through this acoustic window, the lumbar plexus is visualized as a hyperechoic structure located in the posterior part of the psoas muscle (Figure 1) [9].

**Figure 2:**
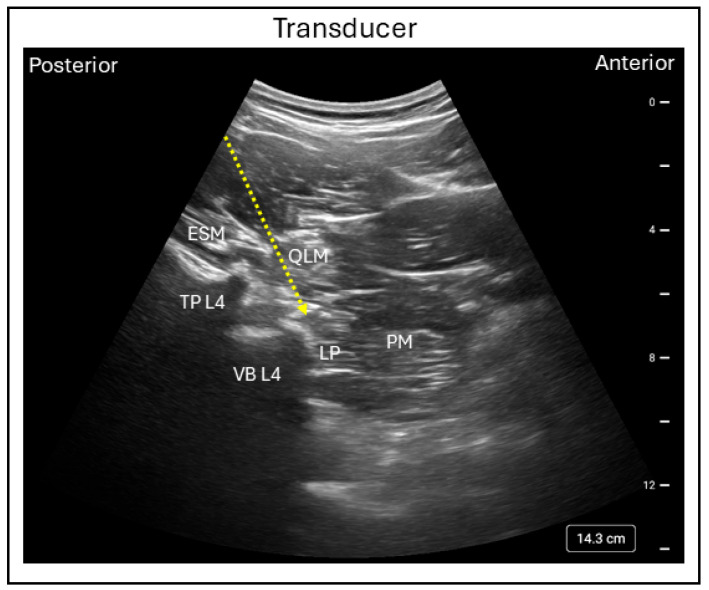
Ultrasound image demonstrating the in-plane Shamrock lumbar plexus block technique using the posterior approach. TPL4 = Transverse process lumbar 4; VBL4 = Vertebral body lumbar 4; LP = Lumbar plexus; ESM = Erector spinae muscle; QLM = Quadratus lumborum muscle; PM = Psoas muscle; Dashed line = Needle trajectory.

In the Shamrock technique, the patient is positioned in the lateral decubitus position with the side to be blocked facing upward and the hips and knees flexed to improve access to the lumbar paravertebral region. A low-frequency curvilinear transducer is placed on the patient’s flank just above the iliac crest in an axial orientation. By sliding the transducer posteriorly, a characteristic “shamrock” (or three-leaf clover) appearance can be visualized, consisting of the L4 transverse process centrally, the psoas major muscle anteriorly, the quadratus lumborum muscle superiorly, and the erector spine muscle posteriorly. Within the posteromedial compartment of the psoas muscle, the lumbar plexus appears as a hyperechoic structure (Figure 2) [10].

In both of these techniques, an in-plane needle insertion is used, directing the needle towards the hyperechoic lumbar plexus under USG. Injection of local anesthetic around the plexus typically produces complete sensory and motor blockade of the femoral and obturator nerves of the ipsilateral side within 30 min, regardless of the type of anesthetic used. The volume of local anesthetic, ranging from 20 mL to 40 mL of 0.5% bupivacaine or ropivacaine, is determined according to the desired spread and patient size, with these doses achieving approximately 50% to 95% success in blocking all three target nerves [11]. The Shamrock and trident techniques provide similar analgesic efficacy; however, the Shamrock approach can be faster to perform and may be more comfortable for the patient [12]. Overall, complications associated with lumbar plexus block are uncommon. The incidence of major complications—such as renal injury, intravascular injection, retroperitoneal hematoma, or unintended spread of local anesthetic via intervertebral foramina—is rare, with an estimated incidence of less than 1%; furthermore, the incidence of peripheral nerve injury following a lumbar plexus block has been reported to be as low as 0.1% [13].

Incomplete analgesia is most commonly attributed to incorrect needle placement, which may result in inadequate spread of the local anesthetic. The variability in patient anatomy and limited visualization of deep structures may further contribute to block failure. The incidence of incomplete analgesia following lumbar plexus block has been estimated to be as high as 15%. The technical difficulty associated with placing a lumbar plexus block has contributed to the growing popularity of superficial fascial plane blocks, which are less challenging to perform and enable better visualization of the surrounding anatomy when compared with deeper plexus blocks.

Although continuous lumbar plexus catheters provide prolonged postoperative analgesia, their placement shares similar technical challenges with single-injection lumbar plexus blocks and is associated with similar risks of block failure and neurologic or vascular complications. In addition, unintended femoral nerve blockade and catheter dislodgement or migration may occur. When placed using the posterior psoas compartment approach, these catheters have been shown to provide prolonged analgesia by maintaining a continuous infusion for 1 to 3 days.

The combination of continuous LPB with 0.5% ropivacaine and a sciatic nerve block can provide surgical anesthesia and postoperative analgesia lasting up to 13 h before the first request for rescue analgesics following TKA [14]. The authors reported that surgical anesthesia was successfully achieved in 78% of patients, with an overall patient satisfaction rate of 92%, suggesting that LPB can be effectively performed in TKA patients and may enhance patient comfort and satisfaction. In a prospective randomized study comparing LPB combined with sciatic nerve block to a femoral–sciatic nerve block combination, the onset of pain [437.5 (70–1150) vs. 1270 (700–1605) min] and time to first analgesic request [609 ± 87.4 vs. 1347.5 ± 62.8 min] was shorter in the LPB group. However, postoperative opioid consumption and pain scores were significantly improved in the LPB group (*p* < 0.05) [15].

In contrast, a pooled data meta-analysis evaluating multiple nerve block techniques for TKA found no significant differences between LPB and placebo in pain sores, opioid consumption, or complications such as nausea and vomiting up to 48 h after surgery [16]. Another meta-analysis investigating the hierarchy of interventions has reported that LPB ranked second to femoral nerve block (FNB), whether administered as a single injection or in combination with other nerve blocks, in terms of analgesic effectiveness. Moreover, LPB was associated with lower rates of nerve injury and block failure compared to FNB [17]. These findings suggest that the variable effectiveness of LPB may be due to limited visibility of the target plexus under ultrasound guidance or catheter migration away from the plexus during block placement and maintenance [18]. Furthermore, a recent cadaver study has demonstrated that local anesthetic spreads in multiple directions, crossing fascial planes from the injection site, which may explain the disproportionate and heterogenous blockade observed among patients [19]. Together, these factors may contribute to the inconsistent clinical effects reported with LPB.

Although TKA is increasingly performed in an outpatient setting, LPB may still be useful in hospitalized patients when multiple nerves must be covered and other targeted blocks are not feasible, especially since its effectiveness has been demonstrated as second only to femoral nerve block.

### 3.2. Fascia Iliaca Block

The fascia iliaca block (FIB) was first described by Dalens et al. in 1989 as an alternative to the three-in-one lumbar plexus block [20]. This novel block was thought to provide similar benefits to the three-in-one block by targeting the femoral, lateral femoral cutaneous, and obturator nerves with a single injection dorsal to the fascia iliaca, rather than at the lumbar plexus itself [21]. The femoral and lateral femoral cutaneous nerves course through the potential space of the fascia iliaca compartment, which lies lateral to the femoral artery, above the iliacus muscle, just proximal to the inguinal region of the upper thigh, and is bounded superolaterally by the iliac crest and medially by the fascia overlying the psoas muscle. The obturator nerve runs more medially through the psoas muscle and can be inconsistently blocked by the fascia iliaca block, depending on the site of injection.

**Figure 3:**
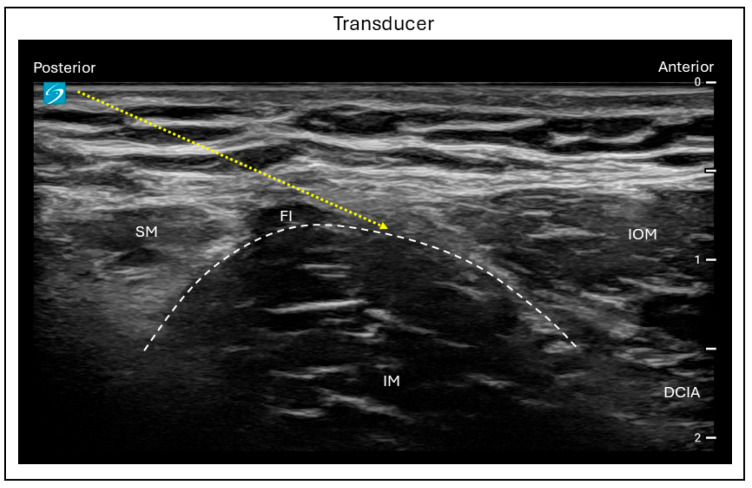
Ultrasound image demonstrating the in-plane suprainguinal fascia iliaca block technique. FI = Fascia iliaca; SM = Sartorius muscle; IM = Iliacus muscle; IOM = Internal oblique muscle; DCIA = Deep circumflex iliac artery; Dashed line = Needle trajectory.

Under USG, the FIB can be performed using either a suprainguinal or infrainguinal technique [21]. For both approaches, the patient lies on their back and a high-frequency linear ultrasound probe is typically used; however, in obese patients, a low-frequency curvilinear probe may provide better visualization. For the suprainguinal approach, the probe is placed in a longitudinal orientation just medial to the anterior superior iliac spine, with a slight rotation toward the umbilicus to allow for a medial scan over the inguinal ligament. The characteristic “bowtie” appearance is visualized, formed cranially by the inferior border of the internal oblique muscle and caudally by the superior border of the sartorius muscle. At approximately 1 cm depth, the fascia iliaca is visualized deep to the sartorius muscle, covering the iliacus muscle. Using an in-plane needle insertion in a caudo-cephalad direction, the facial plane between the fascia iliaca and the iliacus muscle is targeted (Figure 3).

**Figure 4:**
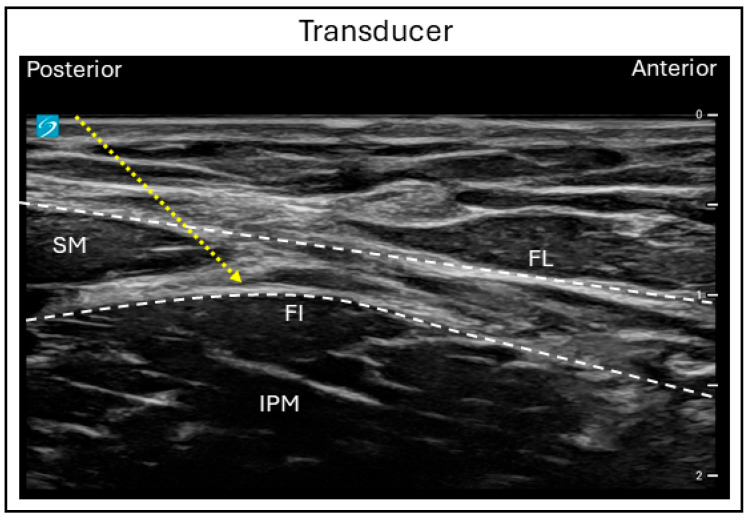
Ultrasound image demonstrating the in-plane infrainguinal fascia iliaca block technique. FL = Fascia lata; FI = Fascia iliaca; SM = Sartorius muscle; IPM = Iliopsoas muscle; Dashed line = Needle trajectory.

For the infrainguinal approach, the probe is initially placed in a transverse orientation below the inguinal ligament to identify the femoral artery, vein, and the nerve. By sliding the probe laterally, the facia iliaca can be visualized as splitting into superficial and deep layers as it encircles the sartorius muscle, with the iliacus muscle lying beneath. The needle is placed in a latero-medial direction, in-plane with the ultrasound probe, and advanced through the fascia lata and fascia iliaca to reach the plane between the fascia iliaca and the iliacus muscle (Figure 4).

In both techniques, hydro-dissection is used to open the plane between the fascia iliaca and iliacus muscle before injection of the local anesthetic. For the suprainguinal approach, 40 mL of local anesthetic is commonly required, whereas 30 mL to 40 mL is needed for the infrainguinal approach to ensure adequate spread to all three target nerves [22]. Either 0.25% or 0.5% bupivacaine or ropivacaine may be used, depending on the patient’s maximum allowable dose for the respective local anesthetic. Quadriceps weakness is a common side effect of the FIB, resulting from spread of the local anesthetic to the femoral nerve. Block failure is another recognized complication, which may occur due to the difficulty of accurately identifying and injecting into the target plane when using a single-injection technique or as a result of catheter migration when a continuous catheter is used.

The analgesic efficacy of the suprainguinal FIB has been shown to be superior to that with the infrainguinal technique during the first 24 h following TKA surgery. Prospective randomized trials have demonstrated lower opioid consumption in patients receiving suprainguinal FIB compared to infrainguinal FIB after TKA [(34.57 ± 33.95 vs. 86.67 ± 34.57 mg, *p* = 0.006) and (131.5 ± 76.8 vs. 201.5 ± 85.1 μg, *p* = 0.001)] [23,24]. Additionally, pain intensity at 6 h postoperatively was significantly lower and the time to first analgesic request was significantly longer in the suprainguinal FIB group. However, the time to place the blocks and complication rates did not differ between techniques. The superior analgesic effect of suprainguinal FIB is likely due to the more consistent spread of local anesthetic to all three target nerves [25]. In a meta-analysis comparing FIB with other nerve block techniques, FIB was associated with the lowest rates of postoperative complications such as nausea, vomiting, and urinary retention. However, no significant differences in pain scores or opioid consumption were observed when compared to placebo up to 48 h after TKA [16]. Nevertheless, FIB demonstrated better effectiveness in reducing pain scores and opioid consumption compared with most other nerve blocks, with the exception of the femoral nerve block, femoral-based combinations, and the LPB [17].

Although continuous FIB can provide prolonged analgesia and reduce opioid consumption without significantly affecting ambulation, catheter-related complications—such as catheter dislodgement and block failure—have been reported in up to 60% of cases [26]. Therefore, single-injection FIB may be considered as an alternative peripheral nerve block within a multimodal analgesic strategy when first-line techniques cannot be performed in patients undergoing TKA in either outpatient or inpatient settings.

### 3.3. Femoral Nerve Block

FNB has evolved from a landmark technique introduced in 1950s to a safe and precise ultrasound-guided procedure used commonly at present. The femoral nerve is a major branch of the lumbar plexus arising from the ventral rami of L2–L4. The nerve travels beneath the inguinal ligament and enters the femoral triangle, where it divides into anterior and posterior branches [27]. The femoral nerve provides sensory innervation to the anterior thigh, knee, and medial lower leg, as well as motor innervation to the quadriceps femoris, sartorius, and pectineus muscles.

**Figure 5:**
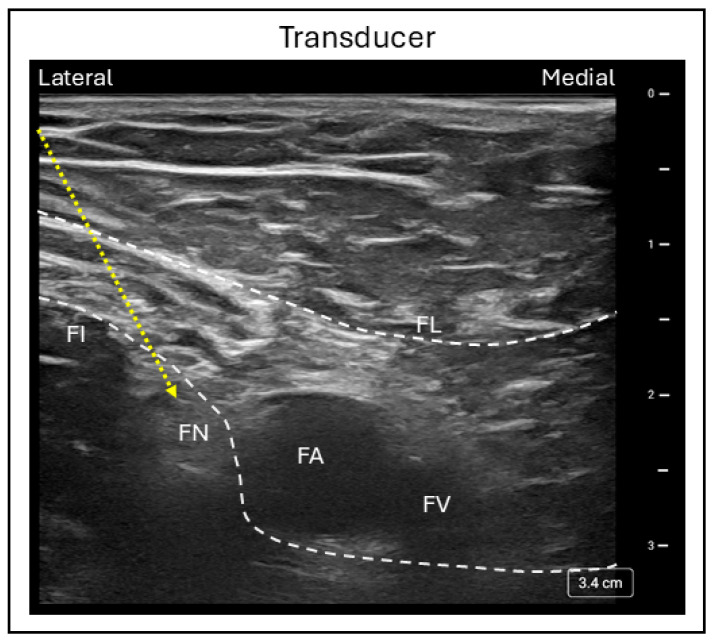
Ultrasound image demonstrating the in-plane femoral nerve block technique. FI = Fascia iliaca; FL = Fascia lata; FN = Femoral nerve; FA = Femoral artery; FV = Femoral vein; Dashed line = Needle trajectory.

An ultrasound-guided FNB is performed with the patient in the supine position and the leg in a neutral or slightly abducted and externally rotated position. A high-frequency curvilinear probe is placed in a transverse position just below the inguinal crease to identify femoral vessels, with the common femoral artery and vein visualized within the femoral sheath. By scanning from medial to lateral along the vessels, the hyperechoic femoral nerve can be identified within the sheath as an oval honeycomb structure. The fascia lata and fascia iliaca are seen superficial to the nerve, while the iliopsoas muscle lies deep with respect to it (Figure 5).

With the in-plane technique, the needle is inserted in a lateral to medial direction and advanced toward the femoral nerve until both the fascia lata and fascia iliaca are pierced. Local anesthetic is then injected adjacent to the nerve, producing a circumferential spread around it [28]. A minimum effective volume of 15 to 22 mL of 0.5% bupivacaine or ropivacaine has been shown to provide adequate postoperative analgesia [29]. Quadriceps weakness is a common and expected side effect of FNB, while nerve injury and vascular puncture are potential complications associated with the procedure.

FNB has been shown to provide effective analgesia after TKA for up to 48 h postoperatively. In a randomized trial using 40 mL of 0.5% bupivacaine, opioid consumption was significantly less when compared to placebo (18.7 vs. 39.6 mg, *p* < 0.001), as were pain scores at rest and movement (*p* < 0.001) during the first 48 h after surgery [30]. A systematic review and meta-analysis comparing single-injection and continuous FNB demonstrated lower pain scores at 24 h with continuous FNB [Standard Mean Difference (SMD), −0.27; 95% CI, −0.50 to −0.05] and reduced opioid consumption (SMD, −1.04; 95% CI, −1.79 to −0.28) up to 48 h after surgery compared to single-injection FNB [31]. These findings support the role of the femoral nerve as a major contributor to knee innervation, with its smaller articular branches supplying the joint [4]. A meta-analysis investigating multiple nerve blocks for TKA found that single-injection FNB significantly reduced pain scores up to 6 h and opioid consumption for up to 24 h compared to placebo, whereas continuous FNB provided superior analgesia compared to other nerve blocks up to 48 h postoperatively [16]. The authors also reported that FNB significantly reduced postsurgical urinary retention rates and was associated with the highest patient satisfaction [16]. Furthermore, a random-effects network meta-analysis demonstrated that FNB combined with additional nerve blocks, such as sciatic and obturator blocks, produced the best postoperative outcomes in terms of pain scores, opioid consumption, and range of motion, whether analyzed individually or in combination [17].

As the analgesic duration and degree of motor weakness from FNB alone can vary with the volume of local anesthetic injected, combining a low-volume FNB with sciatic and obturator nerve blocks may be preferable for TKA patients admitted for 24 h observation, where early ambulation is not essential. Continuous FNB may be considered for patients who require more intensive pain management for 48 h or longer postoperatively. However, catheter-related complications such as migration, obstruction, and leakage are common and should be considered [32,33].

### 3.4. Sciatic Nerve Block

The sciatic nerve originates from the sacral plexus and is composed of nerve roots L4 through S3. The nerve passes deep to the piriformis muscle and exits through the greater sciatic foramen, descending along the thigh before branching near the popliteal fossa into the tibial and common peroneal nerves [34]. Although the sciatic nerve primarily provides motor innervation to the lower extremity, it has long been targeted for analgesia in lower extremity surgeries; this nerve is especially relevant as it contributes significantly to the knee capsule via four to five articular branches [4].

**Figure 6:**
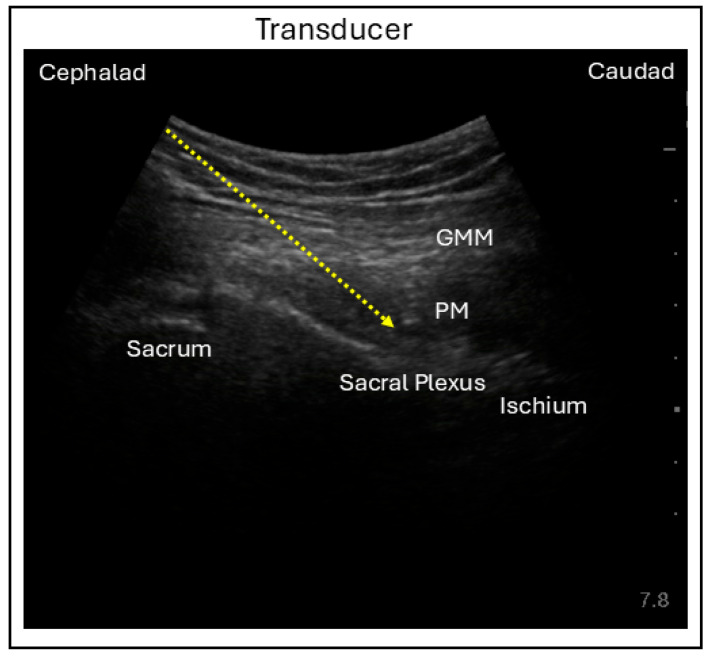
Ultrasound image demonstrating the in-plane lateral parasacral sciatic nerve block technique. GMM = Gluteus maximus muscle; PM = Pyriformis muscle; Dashed line = Needle trajectory.

Various techniques have been described for performing sciatic nerve block (SNB), with proximal approaches including parasacral, transgluteal or subgluteal, and anterior techniques. In the parasacral approach, the patient is positioned in a lateral decubitus position with the operative leg (leg to be blocked) facing upward and both hip and knee flexed. A curvilinear ultrasound probe is placed along the line connecting the posterior superior iliac spine to the greater trochanter. Adjusting the probe distally and medially to reach the level of the greater sciatic foramen, the posteromedial surface of the ischium can be identified with the piriformis superficial to it and sacral plexus underneath it. Using an in-plane needle technique with a lateral to medial direction, the needle is advanced toward the posteromedial end of the ischium for local anesthetic injection and sacral plexus blockade (Figure 6) [35].

**Figure 7:**
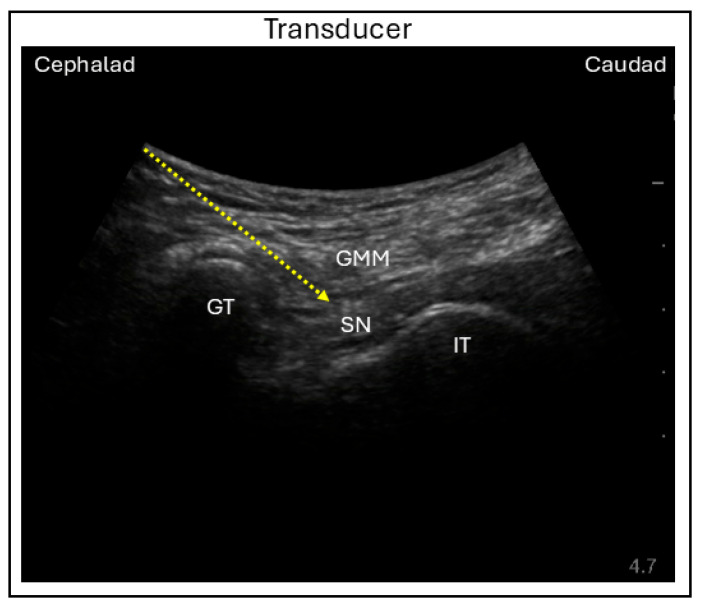
Ultrasound image demonstrating the in-plane lateral subgluteal sciatic nerve block technique. GT = Greater trochanter; GMM = Gluteus maximus muscle; SN = Sciatic nerve; IT = Ischial tuberosity; Dashed line = Needle trajectory.

In the transgluteal or subgluteal approach, with the patient in the same lateral decubitus position, a curvilinear probe is placed along a line connecting the ischial tuberosity and the greater trochanter, either over the gluteus or along the subgluteal crease. The sciatic nerve can be visualized beneath the gluteus maximus and quadriceps femoris, with the head of the biceps femoris appearing medially in the subgluteal view. The sciatic nerve is targeted using an in-plane needle technique with a lateral to medial direction to complete the SNB (Figure 7) [36,37].

**Figure 8:**
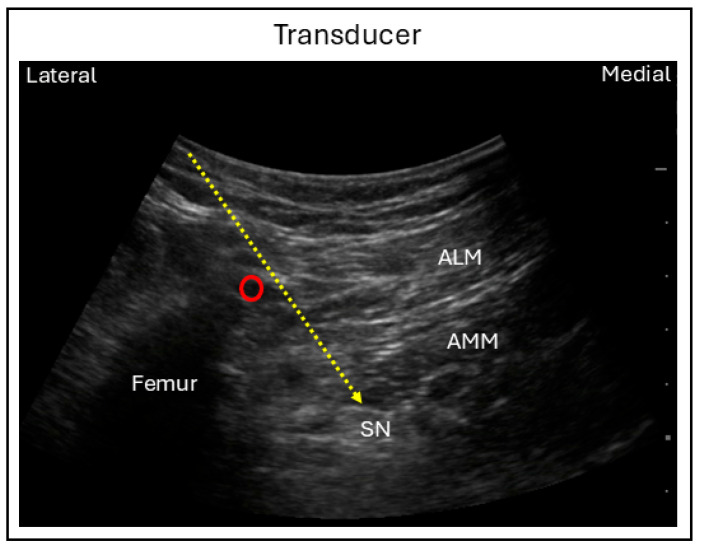
Ultrasound image demonstrating sciatic nerve block using the anterior technique. Red circle = Femoral artery; SN = Sciatic nerve; ALM = Adductor longus muscle; AMM = Adductor magnus muscle.

For the anterior technique, the patient is positioned supine with the hip and knee flexed and the leg externally rotated approximately 45 degrees. A curvilineal ultrasound probe is positioned transversely on the medial thigh just distal to the inguinal crease, in order to visualize the femur and the adductor muscles inserting to the medial aspect of the femur. While the adductor canal is located superficially, the sciatic nerve lies deep to the adductor magnus and posteromedial to the femur. Using an in-plane needle technique, the needle is advanced from anteromedial to posterolateral direction through the adductor muscles to reach the sciatic nerve (Figure 8) [38]. This approach is particularly useful in patients who are unable to lie prone or on their side, as it allows direct access to the sciatic nerve from the anterior aspect of the leg.

**Figure 9:**
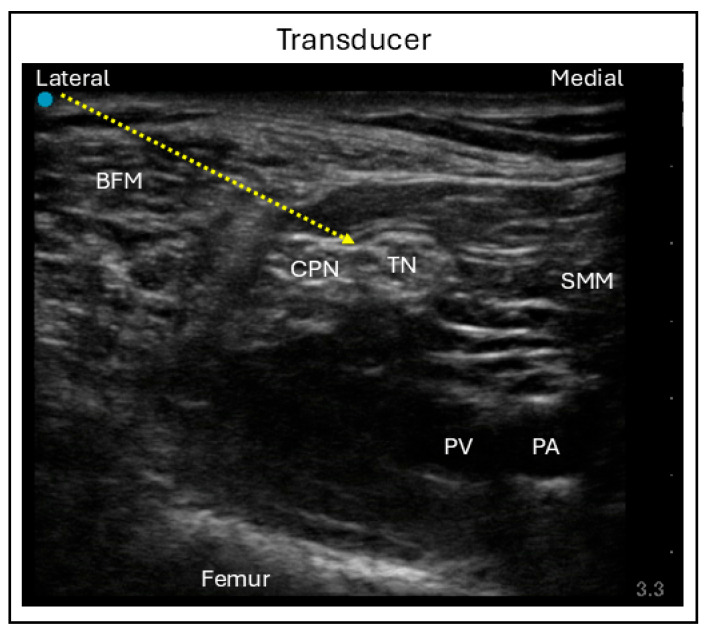
Ultrasound image demonstrating sciatic nerve block using the distal in-plane technique. Dashed line = Needle trajectory; BFM = Biceps femoris muscle; CPN = Common peroneal nerve; TN = Tibial nerve; SMM = Semimembranosus muscle; PA = Popliteal artery; PV = Popliteal vein.

The distal approach, known as the popliteal technique, is performed with the patient in a supine position, the knee flexed, and the leg elevated. A linear ultrasound probe is placed at the level of the popliteal crease to visualize the popliteal artery and the superficially lying popliteal vein. At this level, the divisions of the sciatic nerve—namely, the tibial and common peroneal nerves—can be visualized. Sliding the probe cranially brings these divisions together, allowing the sciatic nerve to be located between the medial semimembranosus muscle lateral biceps femoris muscle. A slight caudal tilt of the probe may be required to optimize visualization of the nerve. Using an in-plane needle technique, the needle is advanced into the perineural space surrounding the sciatic nerve, and local anesthetic is injected to complete the blockade (Figure 9) [39].

The mean volume of local anesthetic required for SNB is approximately 12 mL for the proximal techniques and 20 mL for the distal approach [40], with a concentration of 0.5% bupivacaine or ropivacaine being optimal. Regardless of approach, the most significant drawback of this block is motor weakness, which can affect ambulation and increase the risk of falls. Although rare, nerve injury following sciatic nerve block has been reported slightly more frequently than with some other peripheral nerve blocks, potentially due to anatomy, limited compliance of the perineural sheath, and patient-related factors such as diabetes or pre-existing neuropathy.

Both the proximal and distal SNB approaches have been shown to reduce postoperative pain for up to 8 h following TKA, with a significantly lower proportion of patients experiencing anterior and posterior knee pain compared to placebo (*p* < 0.005) [41]. Although their analgesic efficacy is similar, the distal technique is associated with a shorter procedural time, fewer needle passes, and less patient discomfort compared to the proximal technique. A meta-analysis evaluating different nerve blocks and their combinations for TKA demonstrated that SNB combined with FNB significantly reduced pain scores for up to 6 h and opioid consumption for up to 24 h compared to placebo; however, no differences were observed in functional recovery, nausea, vomiting, and hospital length of stay [16]. Another meta-analysis reported that FNB combined with SNB was the most effective strategy for postoperative analgesia, opioid sparing, and outcomes such as functional recovery and blood loss, when compared to other nerve blocks [17]. However, the authors also reported that this combination was associated with the highest incidence of falls and nerve injury. When performed as a continuous block using 0.2% ropivacaine in combination with continuous FNB, SNB provides superior analgesia with significantly lower pain scores (*p* = 0.035) and greater opioid-sparing effects [4.9 (5.9) vs. 9.7 (9.5) mg, *p* = 0.002], when compared with single-injection SNB combined with FNB [42].

Given the associated risk of motor weakness and falls, the use of SNB in combination with FNB may be most appropriate in carefully selected TKA patients admitted for 24 h, in whom postoperative pain control is prioritized over early ambulation. The use of continuous SNB catheters warrants caution due to the increased risk of falls and the potential for nerve dysfunction compared with other regional techniques for TKA.

### 3.5. Adductor Canal Block

The adductor canal is an anatomical passageway in the medial thigh bordered laterally by the vastus medialis, medially by the adductor longus and adductor magnus, and anteriorly by the vasoadductor membrane. This canal extends from the apex of the femoral triangle proximally to the adductor hiatus distally. Although there are inconsistent reports regarding the neurovascular structures that traverse the adductor canal, the saphenous nerve and the nerve to the vastus medialis (NVM) consistently course within the adductor canal. Both nerves originate from the femoral nerve and contribute sensory innervation to the knee [43]. The saphenous nerve is a pure sensory nerve that provides an articular branch to the anteroinferior knee capsule, whereas the NVM supplies sensory fibers to the knee but continues distally to innervate the vastus medialis muscle to provide motor innervation.

**Figure 10:**
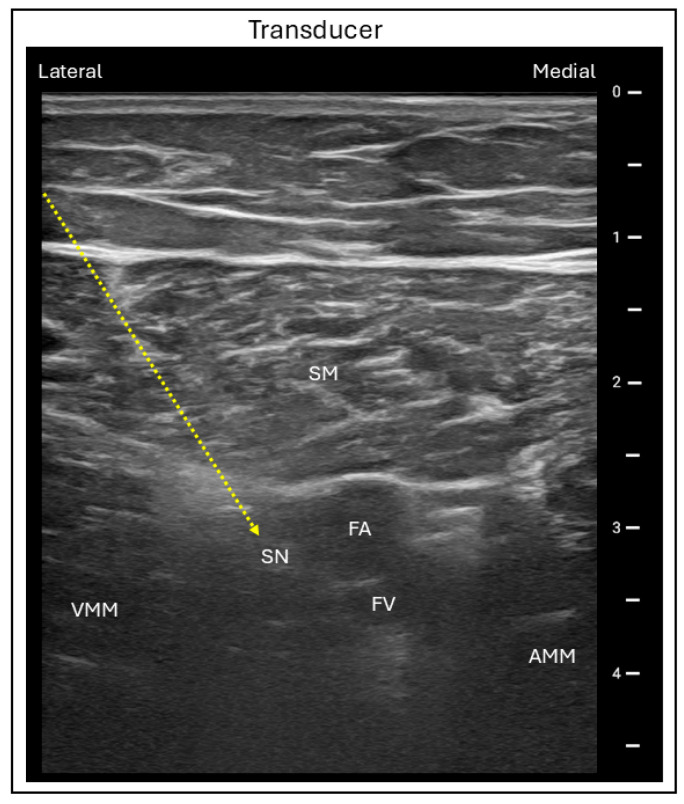
Ultrasound image demonstrating the lateral in-plane technique for adductor canal block. Dashed line = Needle trajectory; AMM = Adductor magnus muscle; SM = Sartorius muscle; VMM = Vastus medialis muscle; SN = Saphenous nerve; FA = Femoral artery; FV = Femoral vein.

Ultrasound-guided adductor canal block (ACB) is conventionally performed at the mid-thigh level with the patient in the supine position and the operative leg slightly abducted and externally rotated, commonly referred to as the “frog-leg” position. A linear ultrasound probe is placed transversely on the anteromedial aspect of the mid-thigh, between the inguinal crease and patella. The femoral artery is first visualized beneath the sartorius muscle, which lies superficially, with the vastus medialis located laterally and the adductor muscles medially and posteriorly. The femoral vein lies posteromedial to the artery, while the saphenous nerve is visualized anterolateral to the artery within the adductor canal. An in-plane needle is then advanced in a lateral to medial direction into the adductor canal, and local anesthetic is injected around the saphenous nerve to complete the block (Figure 10) [44,45]. As the femoral nerve itself is not blocked, the ACB offers the important advantage of preserving quadriceps motor function compared with the FNB.

The minimal effective volumes for ACB performed at the mid and distal portions of the canal have been reported as 10 mL and 20 mL, respectively [46,47], where a concentration of 0.25% or 0.5% bupivacaine or ropivacaine may be used. The primary limitation of ACB is its variable effectiveness, as the duration of analgesia can vary among patients, and motor weakness can occasionally occur due to involvement of the NVM bundle. A multivariate analysis reported a 9% prevalence of quadriceps weakness, which was associated with higher ACB dosing (95% CI, 1.9 to 13.3; *p* = 0.001) [48]. Moreover, continuous catheter placements are often associated with the risk of dislodgment. Regarding the site of blockade within the adductor canal, both proximal and distal ACBs have not demonstrated any differences in pain outcomes involving opioid consumption, although proximal ACB may provide better pain relief for up to 24 h postoperatively [49,50].

The duration of analgesia following single-injection ACB for TKA is approximately 6 h before the first analgesic request, which is significantly longer [365.71 ± 53.7 vs. 150.429 ± 22.5 min (*p* < 0.001)], while opioid consumption is significantly lower [8.628 ± 2.0 vs. 21.91 ± 5.12 mg (*p* < 0.001)] at 24 h after surgery when compared to placebo [51]. This finding was reaffirmed in a pooled analysis comparing ACB combined with periarticular infiltration to other nerve blocks [16]. In addition to demonstrating lower urinary retention rates, ACB was associated with superior functional recovery up to 48 h after surgery and shorter hospital length of stay [15]. However, with regard to analgesic effectiveness, ACB ranked below other proximal nerve blocks and their combinations for TKA [17].

Overall, its analgesic effects can be considered intermediate, following FNB and its combinations. However, comparatively it has the best analgesic efficacy 24 h after TKA when combined with local infiltration, and it is considered the best option for promoting range of motion and reducing length of hospital stay after the surgery. Continuous ACB with 0.2% to 0.5% concentrations of ropivacaine has not been shown to provide superior analgesia or fewer complications when compared with single-injection ACB [52]. Therefore, ACB may be considered an effective and safe analgesia option for patients undergoing TKA in the outpatient setting. However, patients should be informed of the potential risk of motor weakness and possible block failure due to catheter migration, so that they can take necessary precautions during recovery.

### 3.6. Interspace Between Popliteal Artery and Capsule of the Knee Block

The iPACK block, targeting the interspace between the popliteal artery and the knee capsule, is a recently described technique used to provide posterior knee analgesia for surgical patients. First described in 2012, the iPACK block was proposed to anesthetize the terminal articular nerve branches supplying the posterior knee while preserving the sensorimotor function of the sciatic nerve and its tibial and common peroneal branches. Although the innervation of the posterior knee is anatomically complex and can vary between individuals, it is commonly innervated by the posterior articular branches of the sciatic, tibial, common peroneal, and obturator nerves, with the tibial nerve serving as the main contributor [53].

**Figure 11:**
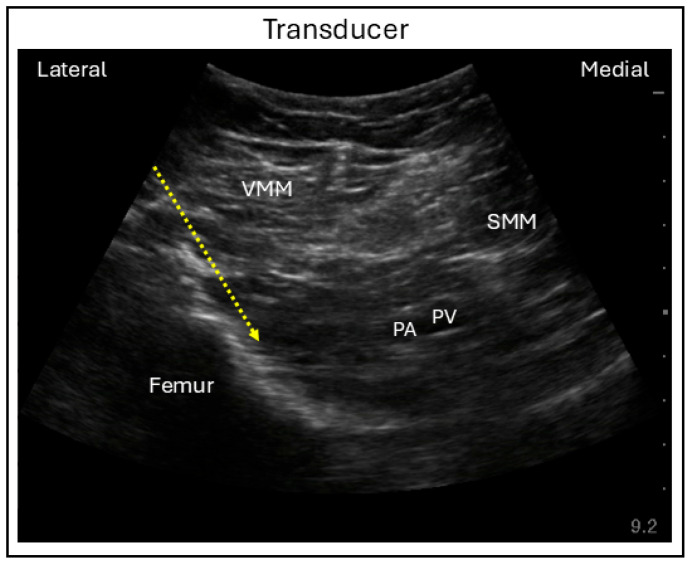
Ultrasound image demonstrating lateral in-plane technique for the interspace between the popliteal artery and the capsule of the knee (iPACK) block. VMM = Vastus medialis muscle; SMM = Semimembranosus muscle; PA = Popliteal artery; PV = Popliteal vein; Dashed line = Needle trajectory.

The ultrasound-guided approach to the iPACK block is relatively simple, performed with the patient supine and the operative leg placed in a frog-leg position. Either a linear or curvilinear transducer probe may be used, depending on the depth of the interspace from the skin surface. The probe is placed a few centimeters proximal to the patella and posteromedial aspect of the thigh to identify the popliteal artery and femoral condyle. To improve visualization, the probe may be moved a few centimeters cranially until the femur shaft and popliteal artery come into view. Using an in-plane lateral to posterior needle approach, the needle is advanced into the interspace between the femur and popliteal artery, where the local anesthetic is deposited (Figure 11) [54].

The minimal effective volume required to achieve a 90% success rate for the iPACK block in patients undergoing TKA has been reported to be 18 mL [55], where either bupivacaine or ropivacaine at a concentration of 0.25% is sufficient. Although the iPACK block is generally considered safe, potential complications include unintended spread of local anesthetic to the sciatic nerve resulting in motor weakness, as well as limited efficacy or block failure due to incorrect injection site or inadequate spread to the posterior plexus of the knee capsule. The iPACK block can be performed either proximally (a finger-breadth from the base of the patella) or distally (at the superior border of the femoral condyles). In a randomized trial, weakness due to common peroneal nerve blockade from both proximal and distal iPACK block approaches lasted 12 h, with no significant difference between them (*p* = 0.76), and was significantly less than that observed with tibial nerve block (*p* = 0.001) [56]. However, studies directly comparing proximal and distal iPACK blocks for postoperative analgesia following TKA remain limited.

When incorporated into a multimodal analgesia regimen that includes ACB and local anesthetic infiltration, the iPACK block demonstrated effective analgesia for up to 12 h, reducing pain scores following TKA [57]. Continuous iPACK block is not practical because the iPACK fascial plane is poorly defined and does not reliably support catheter placement; however, single-injection iPACK block, when combined with ACB, genicular nerve blocks, and local anesthetic infiltration, provides favorable analgesia by reducing pain and improves functional outcomes between 24 and 48 h after TKA. A network meta-analysis of studies investigating several combinations of sensory selective nerve blocks demonstrated that, for 24 to 48 h area-under-the-curve pain scores, iPACK block combined with ACB had the highest P-score probability (89%) of being the most effective for pain control [58]. Similarly, another meta-analysis showed that iPACK block combined with ACB and genicular nerve block (GNB) was most likely to achieve the lowest pain scores and the shortest time to ambulation [59].

These findings support incorporating the iPACK block in combination with other motor-sparing regional techniques, such as ACB, to improve postoperative analgesia and functional recovery in both outpatient and inpatient TKA patients. However, both patients and surgeons should be aware of the potential for associated motor weakness, such that rehabilitation strategies can be optimized and the likelihood of falls decreased.

### 3.7. Genicular Nerve Block

The genicular nerves are terminal articular branches that provide sensory innervation to the knee joint. There are five genicular nerves that have been demonstrated as identifiable using ultrasonography: the superomedial (SMGN), superolateral (SLGN), inferomedial (IMGN), inferolateral (ILGN), and recurrent peroneal genicular nerves. Together, these nerves supply the four quadrants of the knee joint. The SMGN and SLGN typically arise from the femoral nerve and the nerves to the vastus medialis and vastus lateralis, whereas the IMGN, ILGN, and recurrent peroneal genicular nerves originate from the sciatic nerve, tibial nerve, obturator nerve, and common peroneal nerves [60]. Despite variability in their proximal origins, the genicular nerves follow relatively consistent distal courses around the knee, making GNB a useful sensory block for TKA.

**Figure 12:**
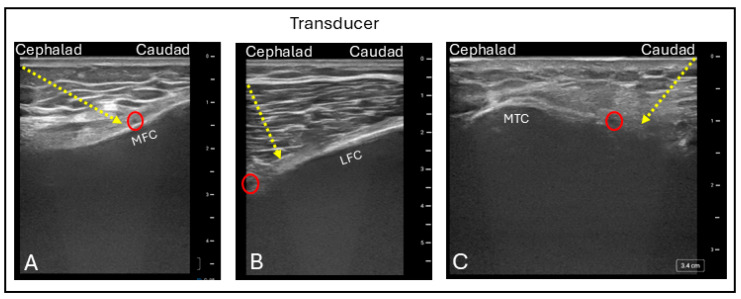
Ultrasound images demonstrating: (**A**) The in-plane superomedial genicular nerve block technique; (**B**) the superolateral genicular nerve block technique; and (**C**) the inferomedial genicular nerve block technique. Red circle = Genicular artery; MFC = Medial femoral condyle; LFC = Lateral femoral condyle; MTC = Medial tibial condyle; Dashed line = needle trajectory.

For ultrasound-guided GNB, the patient is positioned supine with the knee flexed approximately 15 to 30 degrees. A linear probe is placed longitudinally over the distal femur and proximal tibia to identify the femoral and tibial condyles, respectively. Using color Doppler imaging, the genicular arteries are visualized as they course over the bone; the genicular nerves typically lie adjacent to these vessels and may occasionally be directly visualized. The SMGN and SLGN are identified along the medial and lateral aspects of the femoral condyles, respectively, while the IMGN and ILGN are located along the medial and lateral aspects of the tibial condyles. Using an in-plane needle approach, the needle is advanced to the periosteum adjacent to the genicular artery, where local anesthetic is deposited in each quadrant to complete the block (Figure 12) [61]. Blocking of the ILGN is often avoided, due to its close proximity to the common peroneal nerve, in order to minimize the risk of motor weakness.

A dose of 5 mL of local anesthetic at a 0.25% concentration (either bupivacaine or ropivacaine) per nerve, for a total volume of 15 mL targeting the SMGN, SLGN, and IMGN, has been found to ensure effective sensory blockade of the knee joint [62]. In addition to the potential for motor blockade with ILGN block, GNB poses a risk of vascular injury or inadvertent injection due to the close proximity of the genicular arteries.

The analgesic effect of a single-injection GNB including the SMGN, SLGN, and IMGN can last up to 24 h following TKA, with consistently lower pain scores having been reported [63]. On the other hand, a pooled analysis of several studies has shown that combining GNB with ACB and iPACK block provides the most favorable analgesia, offering effective pain control and improved functional outcomes, particularly between 24 and 48 h postoperatively [59,64]. These findings support the combination of GNB with other motor-sparing nerve blocks for patients undergoing TKA, whether discharged home or admitted for 24 h observation.

## 4. Peripheral Nerve Block Strategies

TKA may be performed either on an outpatient or inpatient basis, with discharge timing determined by surgical technique and patient characteristics. Outpatients are typically discharged on the day of surgery after recovery in the post-anesthesia care unit, whereas inpatients are discharged at one or several days following surgery. Therefore, choosing an appropriate peripheral nerve block as part of a multimodal pain management plan is crucial for optimizing postoperative pain control and can directly influence patient recovery (Table 1). This multimodal pain management strategy includes both pharmacological agents and non-pharmacological techniques such as the nerve blocks and periarticular infiltration to align with Enhanced Recovery After Surgery (ERAS) principles that prioritize opioid-sparing analgesia and early mobilization. In these ERAS pathways, regional nerve blocks techniques must be individualized according to discharge plans, rehabilitation goals, and preservation of quadriceps strength. Integrating nerve blocks with periarticular infiltration within structured surgical pathways will enhance both clinical applicability and recovery outcomes.

For outpatient TKA procedures, effective postoperative analgesia is essential to facilitate same-day discharge. In this context, a combination of motor-sparing nerve blocks—such as ACB, iPACK block, and GNB—administered as single injections may be considered. These techniques can provide analgesia lasting up to 48 h, resulting in adequate pain control, early ambulation, immediate discharge following surgery, and allowing for recovery at home [59,64]. In addition, sustained analgesia may encourage patients to begin their active and passive rehabilitation immediately after surgery.

If all three blocks cannot be performed, ACB combined with iPACK block should be considered over ACB with GNB due to the superior analgesic and functional outcomes of the former [64]. If only one block is feasible, a single-injection ACB remains the preferred option, as it provides up to 24 h of analgesia while preserving quadriceps strength, supporting early discharge and rehabilitation benefits such as improved range of motion [16]. While all these motor-sparing nerve blocks are primarily sensory-specific, local anesthetic can occasionally spread to nearby motor branches, potentially causing leg weakness, especially when large volumes are used. Therefore, strict adherence to recommended dosing is essential. Nevertheless, as the incidence of leg weakness is low, typically limited to a few muscles, and does not result in significant lower extremity impairment, these blocks can be safely and effectively used for postoperative pain rehabilitation with appropriate patient and provider education. Continuous ACB for up to 48 h, in combination with single-injection iPACK block and GNB, may further enhance the speed of recovery [59]. However, outpatient use of continuous catheters may be limited by risks such as catheter migration, block failure, and poor availability of resources related to post-discharge follow-up.

For inpatient TKA procedures in which patients are discharged within 24 h of surgery, a combination of single-injection ACB, iPACK block, and GNB may again be considered. Given the duration of analgesia provided by these blocks, this strategy may not only improve postoperative pain control but also facilitates the achievement of rehabilitation goals, especially active movements that predominate during this phase of recovery [65]. A continuous ACB may be considered in this regimen for up to 48 h, in order to further enhance functional recovery [59].

In contrast, for inpatient TKA procedures requiring a hospital stay for multiple days, the choice of nerve block strategy may vary depending on the indication for prolonged hospitalization. When an extended stay is planned to optimize postoperative recovery or manage medical comorbidities, a motor-sparing regional anesthesia approach should continue to be prioritized to achieve key postoperative milestones, including effective analgesia, rehabilitation, and readiness for discharge [59,64]. However, when a prolonged inpatient stay is anticipated and early ambulation is not the primary objective, motor-blocking nerve block strategies may be considered. A combination of single-injection FNB and SNB can provide superior analgesia and facilitate passive rehabilitation, while reducing postoperative complications such as nausea, vomiting, and delirium during the first 48 h after surgery [16,17]. If possible, continuous FNB and SNB are preferred over single-injection techniques, as they offer sustained analgesia and improved functional recovery over a 48 h period [16,42]. If an FNB cannot be performed, LPB or FIB may be considered as alternative regional techniques to support postoperative analgesia and recovery goals [14,16,23,24].

## 5. Conclusions

Effective postoperative pain management following TKA is essential for patient comfort, early mobilization, and minimizing opioid consumption. Peripheral nerve blocks remain a cornerstone of multimodal analgesia, providing targeted pain relief with varying effects on motor function and duration of action. Traditionally, femoral nerve blocks and femoral–sciatic–obturator combinations have provided robust analgesia but are limited by quadriceps weakness and an increased risk of falls. Lumbar plexus and fascia iliaca blocks serve as alternatives when broader plexus coverage is needed, particularly in inpatient settings or high-pain scenarios.

In contrast, newer motor-sparing techniques such as the ACB, iPACK block, and GNB have gained preference in enhanced recovery protocols for outpatient TKA, providing effective analgesia while preserving ambulation. Evidence increasingly supports combining ACB with iPACK block and GNB to optimize pain control and functional recovery during the first 24 to 48 h postoperatively. Continuous catheter techniques may further extend analgesia in admitted patients, although they are associated with higher rates of dislodgement and variable reliability depending on the block type.

In summary, a combination of peripheral nerve blocks should be preferred over an individual nerve block for TKA, where the choice of combination should be individualized based on the surgical setting, rehabilitation goals, and patient comorbidities. By prioritizing patient-reported outcomes, functional recovery, and long-term safety, multimodal analgesia strategies that incorporate motor-sparing nerve blocks are expected to continue to evolve toward optimal postoperative care for TKA patients.

## Figures and Tables

**Table 1:** Peripheral nerve block strategies for TKA across postoperative discharge pathways.

Clinical Scenario	Peripheral Nerve Block Strategy	Post Surgical Window	Post-Surgical Milestones
Outpatient TKA (Hospital discharge immediately after surgery)	ACB + iPACK + GNB	48 h	Adequate pain control, early ambulation, immediate discharge, home recovery, early rehabilitation
	ACB + iPACK > ACB + GNB	24–48 h	Pain control, early ambulation, immediate discharge, home recovery, early rehabilitation
	ACB	24 h	Pain control, early discharge, improved range of movements
	Continuous ACB can be considered to augment functional recovery		
Inpatient TKA (Hospital discharge within a day after surgery)	ACB + iPACK + GNB	48 h	Pain control, early ambulation, early discharge, home recovery, improved rehabilitation
	Continuous ACB can be considered to augment functional recovery		
Inpatient TKA (Hospital discharge > 1 day after surgery for optimization of recovery or comorbidities)	ACB + iPACK + GNB	48 h	Pain control, improved ambulation, improved rehabilitation, discharge
	Continuous ACB can be considered to augment functional recovery		
Inpatient TKA (Hospital discharge > 1 day after surgery for optimization of recovery where early ambulation is not a priority)	FNB + SNB	48 h	Pain control, improved rehabilitation, reduced post- surgical complications
	Continuous FNB and SNB can be considered to augment analgesia and functional recovery LPB and FIB can be considered as alternatives when FNB cannot be performed		

## Data Availability

No new data were created or analyzed in this study. Data sharing is not applicable to this article.

## Abbreviations

The following abbreviations are used in this manuscript:

ACB	Adductor canal block
ALM	Adductor longus muscle
AMM	Adductor magnus muscle
AW	Acoustic window
BFM	Biceps femoris muscle
CI	Confidence Interval
CPN	Common peroneal nerve
DCIA	Deep circumflex iliac artery
ESM	Erector spinae muscle
FA	Femoral artery
FI	Fascia iliaca
FIB	Fascia iliaca block
FL	Fascia lata
FNB	Femoral nerve block
FV	Femoral vein
GMM	Gluteus maximus muscle
GNB	Genicular nerve block
GT	Greater trochanter
ILGN	Inferolateral genicular nerve
IM	Iliacus muscle
IMGN	Inferomedial genicular nerve
IOM	Internal oblique muscle
iPACK	Interspace between the popliteal artery and the capsule of the knee
IPM	Iliopsoas muscle
IT	Ischial tuberosity
LFC	Lateral femoral condyle
LPB	Lumbar plexus block
MFC	Medial tibial condyle
NVM	Nerve to the vastus medialis
PA	Popliteal artery
PV	Popliteal vein
PM	Psoas muscle
QLM	Quadratus lumborum muscle
SLGN	Superolateral genicular nerve
SM	Sartorius muscle
SMD	Standard mean difference
SMGN	Superomedial genicular nerve
SMM	Semimembranosus muscle
SN	Sciatic nerve
SNB	Sciatic nerve block
TKA	Total Knee Arthroplasty
TN	Tibial nerve
USG	Ultrasound-guided
VMM	Vastus medialis muscle
